# Synthesis of Bioactive Materials by In Situ One-Step Direct Loading of *Syzygium aromaticum* Essential Oil into Chitosan-Based Hydrogels

**DOI:** 10.3390/gels8040225

**Published:** 2022-04-06

**Authors:** Elena Stoleru, Raluca P. Dumitriu, Gabriela-Liliana Ailiesei, Catalina Yilmaz, Mihai Brebu

**Affiliations:** “Petru Poni” Institute of Macromolecular Chemistry, 41 A Grigore Ghica Voda Alley, 700487 Iaşi, Romania; rdumi@icmpp.ro (R.P.D.); gdarvaru@icmpp.ro (G.-L.A.); duncaty@gmail.com (C.Y.)

**Keywords:** ultrasound emulsification, gelation inducing agent, clove essential oil, oil loaded hydrogels, polysaccharide, nanostructured morphology

## Abstract

Hydrogel conjugates based on chitosan and an essential oil were synthetized by an ultrasound-assisted emulsification approach. Rheology studies revealed a gel-type structure with pronounced compactness and flexibility while SEM showed the formation of a two-level ordered network with highly interconnected pores. The swelling studies indicated a pH-dependent behavior with a significant overshooting effect. The synergistic effects of the components in clove essential oil led to a strong antioxidant character and an enhanced antimicrobial activity of the conjugate hydrogels. The bioactivity was maintained for 6 months, despite a slight decrease in the antimicrobial effect. Hydrogel conjugates were found to be very stable even after two months immersed in acidic solutions that would otherwise dissolve the chitosan matrix. Ultrasound emulsification was proved as an efficient one-step loading method of hydrophobic clove essential oil into hydrophilic chitosan matrix. It was found that clove oil and its components have a double role. Besides providing bioactivity, they also behave as gelation-inducing agents, acting as an alternative to the classical chemical cross-linkers to ensure the good physical and chemical stabilization of chitosan.

## 1. Introduction

Even if the term hydrogel was mentioned for the first time more than one hundred and twenty years ago, in 1894, referring to a colloidal gel, the first application of hydrogels in the biomedical field was reported decades later in 1960 by Wichterle and Lim [1]. Although it has long been a topic of interest to the scientific community and industry, there is still a growing focus on this area due to the versatility of these structures and due to the fact that they have not yet reached the high level of expectations in various domains. Generally, hydrogels are defined as three-dimensional networks of polymeric chains that are chemically or physically cross-linked, insoluble in water but with a great ability to uptake large volumes of water [2]. A very important feature of most hydrogels is their good biocompatibility, which relies mainly on the high-water content that determines the rubbery and soft properties, as well as on the overall physicochemical resemblance to the native extracellular matrix (ECM) [3]. At the moment, the research interests in this regard are focused towards developing hydrogels with tunable properties, desired shapes, suitable porosity, a controlled release of active principles, and an adequate degradation rate [4].

Lately, extensive research has focused on a new generation of hydrogel-based materials with the suitable bioactivity required by a wide range of applications in the biomedical field [5,6,7]. In general, bioactivity is imparted to hydrogels by incorporation into the matrix of compounds with specific functions. Usually, the embedding of the bioactive agents can be performed in two ways, namely by post-loading or by in situ loading [8]. Post-loading methods involve the swelling of hydrogels in a solution of a bioactive agent until equilibrium swelling is reached. By contrast, in situ loading implies mixing the bioactive agent with the precursor polymer solution and the entrapment of the active substance within the matrix occurs during gelation [9].

Various types of bioactive agents such as drugs, antioxidant and antimicrobial compounds, enzymes, cells, etc., can be loaded into hydrogels to confer target activities or functions to them [10,11,12]. Hydrogels generally have a hydrophilic character; thus, they are more efficient for loading hydrophilic agents [13] but they are less efficient, for hydrophobic ones, and therefore have many limitations in this area. The release into a hydrophilic physiological medium of hydrophobic compounds from hydrophilic hydrogels is also challenging, usually involving the alteration or even the degradation of the loading matrix [3]. However, numerous bioactive agents have a hydrophobic nature; therefore, the actual research is geared towards new approaches for incorporating hydrophobic compounds into hydrogels [14].

Among various natural polymers, chitosan, which is the second most plentiful natural polysaccharide next to cellulose, presents a great potential for the synthesis of hydrogel-based materials [15,16]. Due to its versatile properties (cationic, polyelectrolyte, pH responsive) extensive research has been conducted to develop new chitosan-based hydrogels [17,18]. The predominant hydrophilic character of chitosan and its susceptibility to degradation by human enzymes induce biocompatibility and biodegradability, which are valuable properties in the biomedical field [19,20]. Chitosan has a slight antioxidant activity [21] and moderate antimicrobial properties with a narrow spectrum of action [22]. This has to be improved for applications in wound management, tissue engineering and bioresorbable implants, where microbial contamination and the increased microbial resistance to synthetic drugs are among the major threats [23]. Chitosan is one of the few polysaccharides that contain both polar and non-polar fragments in its structure: the hydrophilic D-glucosamine structural units and the hydrophobic N-acetylated residues [24]. Its moderate amphiphilic nature determines the potential to be used in developing emulsion-based systems; thus, it has the ability to load hydrophobic active compounds [25,26].

Recent attention has been paid to the use of essential oils (EOs) as bioactive agents in many domains such as pharmaceutics [27], food-related applications [28,29], cosmetics [30], etc. The main benefits that EOs bring are the antioxidant and antimicrobial activities, and the antiviral, insecticidal, analgesic, anti-inflammatory properties, etc. [31]. Essential oils have complex and particular compositions based mainly on terpenes and terpenoids, sesquiterpenes, phenolic compounds, and aldehydes [32]. The chemical constituents of EOs determine their hydrophobic nature, which may be considered both as an advantage and a limitation, depending on the intended area of application [33]. Based on various studies, essential oils present great potential in combating the antimicrobial resistance of microbes. This potential is assigned to their complex chemical and structural variance, which prevents bacteria developing resistance. The biological effects of EOs do not rely only on the major compounds but mainly on the synergism between all constituents, with the function of the main components regulated by the minor ones [34].

As generally recognized, hydrogels are predominantly hydrophilic; hence, this property strongly restricts their direct loading with essential oils that have a hydrophobic character. A promising strategy to overcome this limitation is represented by the emulsification-based approach [35]. Emulsifiers are generally used to stabilize the dispersion of hydrophilic and hydrophobic coexisting phases, depending on the nature of the components. For example, polysorbate 80, also known as Tween 80, a non-ionic surfactant, is extensively used in the development of stable emulsions for cosmetic, pharmaceutical, and food applications. Tween 80 has a HLB (Hydrophile–Lipophile Balance) value of 15.0, which recommended it for obtaining oil-in-water emulsions [36]. Tween 80 was selected to disperse the clove essential oil into an aqueous chitosan phase based on a previous promising study when stable emulsions were obtained and applied further as polymeric coatings. The ultrasound-assisted emulsification is a technique widely used to produce stable emulsions since sound waves are generally considered safe, non-toxic, and environmentally friendly. Moreover, ultrasounds may contribute to the development of materials with unique properties and functionality [37].

The premises of this research were based on our previous results where functional chitosan-based coatings loaded with clove essential oil (CEO) were obtained and tested as bioactive food packaging materials [23]. The aim of the study was the in situ loading of hydrophobic clove essential oil into a hydrophilic chitosan matrix to obtain hydrogels with improved antimicrobial and antioxidant activity without the supplementary use of crosslinkers. Ultrasound-assisted emulsification was tested as a new gelation method compared with the classical approach using trisodium citrate. The obtained oil-loaded hydrogels were evaluated in terms of morphology, structure, and composition, as well as pH-dependent swelling ability. An in depth rheological characterization was performed to evaluate the viscoelastic properties which are a measure of the spatial arrangement and flexibility of the macromolecular chains. Moreover, the radical scavenging ability and antimicrobial activity of chitosan-based hydrogels were also tested.

## 2. Materials and Methods

### 2.1. Materials

High molecular weight chitosan (CHH) (Mw = 310,000–375,000 g/mol) from crab shells, with 87.8% deacetylation degree as previously determined by ^1^H-NMR spectroscopy [38] and a dynamic viscosity of 400 MPa·s in 1% acetic acid solution, Tween 80 (T80) emulsifier and tri-sodium citrate dihydrate (Cit) ionic cross-linker were purchased from Sigma-Aldrich, Steinheim, Germany. Clove essential oil (CEO), extracted from the buds of *Syzygium aromaticum*, was acquired from Fares, Orăștie, Romania. This oil was selected among other essential oils based on our previous study [23], which revealed a high antioxidant activity with a half maximal inhibitory concentration (IC50) of 8 μg/mL against DPPH radical. The chemical composition of clove essential oil was previously determined by gas chromatography (GC) analysis, with qualitative identification based on mass spectrometry detection and quantitative determination based on flame ionization detection [39]. It was found that CEO consists mainly of eugenol (85.7 wt%), eugenol acetate (7.9 wt%), β-caryophyllene (4.5 wt%), α-caryophyllene (0.9 wt%), and of very low amounts (less than 1 wt%) of numerous compounds such as β-cymene, γ-terpinene, chavicol, thymol, copaene, α-farnesene, and caryophyllene oxide. Eugenol (Eu) of 99% purity, as a model compound for CEO, was purchased from Sigma-Aldrich, Steinheim, Germany. Acetic acid and methanol solvents were of analytical grade, purchased from Chemical Company, Iași, Romania.

### 2.2. Emulsion Formulation and Hydrogel Synthesis

A 1.3% *w*/*v* chitosan solution was prepared by dissolution in 2% *w*/*v* acetic acid and magnetic stirring for 48 h at a speed of 250 rpm. A mixture of chitosan solution (20 mL), Tween 80 (0.018 g) and CEO (150 µL) was homogenized into a 50 mL beaker by the ultrasound-assisted emulsification approach, using an UP50H ultrasonic processor (Hielscher—Ultrasound Technology, Teltow, Germany) at a power of 50 W and 30 kHz frequency for 15 min. The tip of the ultrasound probe was centrally positioned at about 3 cm from the bottom of the beaker. The stabilized emulsions were poured on glass plates, frozen at −20 °C for 2 days and freeze dried for 48 h at −52 °C and 0.13 mbar to obtain CEO-loaded xerogels of about 3 cm in diameter. Classical crosslinking was comparatively performed by immersion for 4 h at 22 °C in 100 mL aqueous solution of 5% *w*/*v* tri-sodium citrate at pH 5.0. The citrate crosslinked hydrogels (CHH/T80/CEO-Cit) were extensively rinsed with distilled water, transferred to a glass plate, and freeze-dried to a constant weight. A reference unloaded xerogel was prepared in similar conditions based solely on chitosan and emulsifier (CHH/T80). While hydrogels are generally recognized as tridimensional polymeric networks able to uptake large amounts of water, there is no clear differentiation in terminology between the dried and hydrated forms of the materials. In this regard, the term “hydrogel” was selected here to define the solid materials in their hydrated form while “xerogel” was used for the freeze-dried form.

### 2.3. Methods

The stability of the xerogels was evaluated by visual inspection of the physical appearance and by solubilization tests through immersion for 3 months in mild acidic HCl solution of pH 3.6 (mimicking the gastric fluid pH) and in strong acidic media of aqueous 10% acetic acid (with high affinity for dissolving chitosan). The properties of the obtained materials, both in dried and in hydrated form, were evaluated by specific methods as described below.

#### 2.3.1. Gas Chromatography Analysis

The release of the main compounds in clove essential oil from the loaded xerogels was determined using a 6890 gas chromatograph coupled with a 5975 Inert XL mass spectrometry detector (Agilent, Santa Clara, CA, USA). The chromatographic separation was performed on a TR-520232 Teknokroma capillary column (crosslinked 95% dimethyl-5% diphenyl-polysiloxane, 30 m × 250 m × 0.25 μm), under a 1 mL/min helium flow, with the inlet heated at 175 °C, and a 100:1 split ratio. The temperature program was started at 60 °C, followed by heating with a rate of 7 °C/min up to 200 °C then of 15 °C/min up to 300 °C which was isothermally maintained for 3.33 min. The qualitative identification of the compounds was performed based on NIST14 spectral database, with a minimum quality of recognition of 80%, and confirmed from Kovats retention indices that were calculated based on a reference mixture of C6–C20 normal paraffins.

#### 2.3.2. Scanning Electron Microscopy

The morphology of hydrogels at the surface and in the cross-section was evaluated by scanning electron microscopy (SEM) on an electron microscope VEGA II TESCAN (Brno, Czech Republic), without any preliminary treatment.

#### 2.3.3. FTIR and ^1^H-NMR Spectroscopy

Clove essential oil and chitosan-based xerogels were comparatively analyzed by FTIR spectroscopy to determine the interactions between the components. A VERTEX 70 spectrometer (Bruker Optics, Ettlingen, Germany) was used to record the Fourier-transform infrared spectra (FTIR) in the 600–4000 cm^−1^ domain, at a spectral resolution of 4 cm^−1^ by co-adding 64 scans. The Attenuated Total Reflectance (ATR) sampling technique was applied, and the instrument was equipped with a ZnSe crystal at a 45° angle of incidence. Prior to each measurement a background spectrum was recorded. The processing of the spectra was achieved using OPUS software version 7.0.

The effect of ultrasonic waves on the reactivity of clove essential oil in 2% *v*/*v* acetic acid solutions was evaluated by ^1^H-NMR spectroscopy using eugenol as a model compound, alone or mixed with Tween 80 surfactant, and recording the spectra in CDCl_3_. ^1^H-NMR spectra were also recorded for the powdered unloaded chitosan sample solubilized in D_2_O/DCl (CHH sample) and for the chitosan–clove oil hydrogel finely dispersed in D_2_O/CD_3_COOD (CHH/T80/CEO sample). The NMR spectra were recorded on a Bruker Avance NEO 400 MHz spectrometer (Germany Rheinstetten, Bruker BioSpin), operating at 400.1 MHz. ^1^H NMR spectra were recorded with a 5 mm four nuclei direct detection z-gradient probe using standard pulse sequences as delivered by Bruker with TopSpin 4.0.8 spectrometer control and processing software. Chemical shifts were reported in δ units (ppm) and were referenced to the sodium 3-(trimethylsilyl)-[2,2,3,3-d_4_]-1-propionate (TSP) internal standard at 0.0 ppm.

#### 2.3.4. Rheological Studies

Rheological investigations were performed using an Anton Paar MCR301 Rheometer (Berlin, Germany) at different shear rates and angular frequencies using a cone-plate geometry measuring system with a cone angle of 1° and a diameter of 50 mm. Prior to each measurement the unloaded and clove oil-loaded hydrogels were placed onto the plate and rested for 5 min to eliminate residual shear history. The flow behavior was tested in the rotational controlled shear rate condition (CSR), where the viscosity (η) of the samples was measured as a function of the increasing shear rate in the 0.1–1000 s^−1^ range. The viscoelastic behavior was evaluated by oscillatory tests, measuring the storage (G′) and loss (G″) dynamic moduli as a function of the angular frequency, ω, in the 0.1–1000 rad·s^−1^ interval. To perform the frequency sweep tests, the linear viscoelastic range of the samples (LVE) was obtained from amplitude sweep tests, with a strain amplitude between 0.01% and 500%, and a constant angular frequency of 10 s^−1^.

#### 2.3.5. Swelling Studies

Dynamic swelling experiments were conducted at different pH values to analyze the behavior of hydrogels in simulated physiological media (e.g., gastric, or intestinal fluids). A phosphate saline buffer solution (PBS) 0.1 M of pH 7.4 and acidic aqueous solutions of pH 3.6 and of pH 5.6 adjusted by HCl 0.1 M were used as swelling media. All solutions were prepared with twice distilled water. Xerogels were immersed in glass vials containing 15 mL of the respective swelling medium at room temperature (~23 ± 1 °C). Then the swollen hydrogels were taken out at regular time intervals, wiped with filter paper to remove excess surface water, and weighed immediately. The swelling degree was determined gravimetrically, as the weight gained by the hydrogel sample over time, according to Equation (1):

(1) $$S=\frac{\left(W_{s}-W_{d}\right)}{W_{d}}\times 100\;\left(\%\right)$$

where *W_s_* is the weight of the swollen hydrogel at time *t*, and *W_d_* is the weight of the hydrogel in the initial dry state.

#### 2.3.6. Antioxidant Activity

The antioxidant activity of unloaded and of oil-loaded chitosan xerogels was evaluated by testing the radical scavenging potential of chitosan-based xerogels against 2,2-diphenyl-1-picrylhydrazyl (DPPH) radical, following an adapted procedure previously reported [40]. Briefly, the procedure involved adding about 16 mg of freeze-dried hydrogels to 6 mL of 0.05 mM DPPH ethanolic solution. After 30 min incubation in dark, a particular volume of the solution was withdrawn from the vessel and the UV-Vis spectrum was recorded with a Cary 60 UV-Vis spectrophotometer (Agilent Technologies, Santa Clara, CA, USA) against ethanol as a blank sample. The solution was clear; thus, no filtering was required before analysis. The DPPH radical in its free form has a strong purple color and by reaction with a proton-donating compound turns its color to light yellow. The color fading of the DPPH solution was quantified by the decrease in absorbance at 517 nm; this is directly correlated with the antioxidant capacity. The antioxidant potential of the xerogels was expressed as the percentage radical scavenging activity (RSA%) calculated using Equation (2):

RSA (%) = [(A_control_ − A_sample_)/A_control_] × 100 (2)

where A_sample_ is the absorbance of DPPH solution incubated with the chitosan-based xerogels with or without clove oil and A_control_ is the absorbance of control (reference DPPH solution).

The experiments were performed in triplicate and the results were expressed as mean values along with their calculated standard deviations.

#### 2.3.7. In Vitro Antimicrobial Activity

Antibacterial and antifungal activities were evaluated at the Veterinary Health and Food Safety Agency (DSVSA) of Iași, Romania, using standard methods approved by the European Committee for Standardization (CEN). Two bacterial strains from American Type Culture Collection, specifically *Staphylococcus aureus* (ATCC 25923)—Gram positive, and *Escherichia coli* (ATCC 25922)—Gram-negative, and one fungal strain, namely *Candida albicans* (ATCC 90028) were used in this study. Prior to the tests, suspensions of bacterial and fungal strains were prepared in peptone saline with a turbidity of 1° McFarland. Afterwards, dilutions were performed until a final concentration of about 1.5 × 10^3^ colony was reached forming units/mL (CFU/mL). From this diluted microbial suspension, 0.1 mL was used to contaminate the hydrogel-based samples and cell density was determined before incubation for 24 h at 25 °C in the dark. After this period, the inoculum was collected using a sterile swab soaked in peptone saline, seeded on the surface of the specific environment (Violet Red Bile Glucose-VRBG agar for *E. coli*, Mannitol Salt Agar-MSA for *S. aureus*, and Sabouraud agar for *C. albicans*) incubated for another 24 h at 37 ± 1 °C, and after that cell density was measured. Growth of microbial cells was determined by finding the difference of the pre-incubation value from the post-incubation value. Microbial cells in specific broth without the presence of the polymeric samples were used as the control. Antimicrobial activity is expressed as a percentage inhibition, which was obtained using Equation (3):

Percentage inhibition = [(C_control_ − C_sample_) × 100]/C_control_ (3)

where C_control_ represents the microbial cell density of the inoculum in the absence of the sample and C_sample_ represents the microbial cell density of the inoculum kept in contact with the polymeric xerogels (loaded or unloaded with oil).

The experiments were performed in duplicate and the results were expressed as mean values along with their calculated standard deviations.

## 3. Results and Discussion

### 3.1. Visual Aspects and the Stability Evaluation of Emulsions and Xerogels

A qualitative evaluation of the visual aspects and the stability in time of emulsions and xerogels offers important information on the interactions between the components in the complex system containing chitosan, tween 80 and clove essential oil.

#### 3.1.1. Evaluation of Emulsions

**Figure 1:**
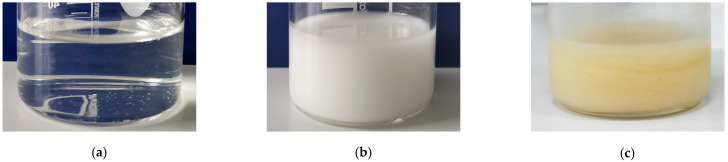
The visual aspect of the chitosan-based solution (**a**), fresh emulsion (**b**) and six month aged emulsion (**c**).

Chitosan completely dissolved in aqueous acetic acid, forming a clear and transparent solution, whose aspect did not change with the addition of the Tween 80 emulsifier, as shown in Figure 1a. However, when clove essential oil was added in the system, an emulsion with a milky appearance was formed (Figure 1b). This remained stable, without phase separation, even after 6 months of on-shelf storage (Figure 1c). This is a sign of very stable interactions among emulsion components. A pale-yellow coloration appeared, most probably due to the ageing of sensible components of CEO oil.

Various clove oil and eugenol-based mixtures in 2% *v*/*v* acetic acid aqueous solution were tested under different conditions in an attempt to obtain insights on how the type of homogenization and composition influenced the interactions between the components in the system.

**Figure 2:**
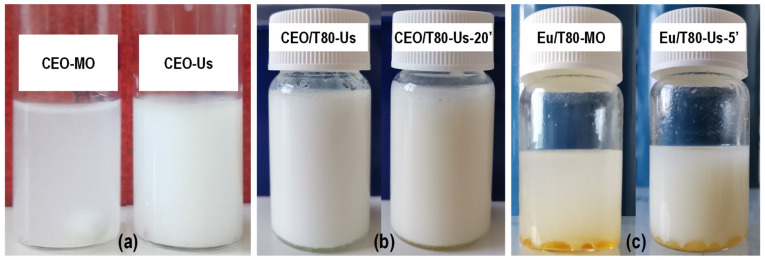
The visual aspect of (**a**) clove essential oil (CEO) dispersions in acetic acid solutions obtained without emulsifier by mechanical homogenization (CEO-MO) or ultrasonication (CEO-US); (**b**) CEO dispersions with emulsifier Tween 80 immediately (CEO/T80-Us) and after 20 min (CEO/T80-Us-20’) from ultrasonication; and (**c**) eugenol (Eu) dispersions in acetic acid solution obtained with emulsifier by mechanical homogenization (Eu/T80-MO) or ultrasonication (Eu/T80-Us-5’).

The dispersion of clove essential oil, generally known as a hydrophobic complex mixture, into emulsifier-free acetic acid solution was evaluated after mechanical and ultrasound homogenization (Figure 2a). It was observed that mechanical homogenization by magnetic stirring (CEO-MO) led to a translucent dispersion with a slightly milky aspect for which phase separation started to be observed within five minutes. However, complete phase separation was not achieved even after a long period of resting, suggesting that some very weak interactions occurred between several components from clove essential oil and the acetic acid solution. Ultrasonication improved the dispersion of CEO as much finer droplets (CEO-Us) that gave an opaque appearance and a more intense white color to the dispersion, which remained stable for about 10–15 min before phase separation could be noticed. Interactions between the components were enhanced by ultrasonication, but this was not enough to stabilize the emulsion.

Adding Tween 80 emulsifier enhanced the dispersion of CEO into the aqueous media (CEO/T80), leading to an emulsion for which a slight phase separation could be observed at the bottom of the flask after about 20 min (Figure 2b). This ensured a sufficient stability window for complete freezing at −20 °C before lyophilization to obtain hydrogels.

Dispersion was also tested on eugenol, the major component in clove essential oil, which was used as the model compound (Figure 2c). Intense shaking slightly dispersed eugenol in the continuous phase, but most parts remained as droplets at the bottom of the flask. Ultrasonication favors dispersion, but could not ensure stabilization in the emulsion: phase separation occurred at a high ratio in less than 5 min. This is an indication that the minor components of clove oil played an essential role in interactions with the aqueous phase towards the development of a relatively stable dispersion.

#### 3.1.2. Xerogels Evaluation

**Figure 3:**
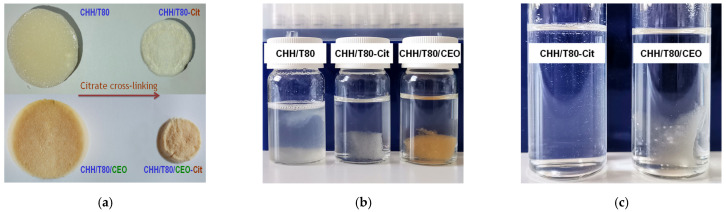
The visual aspect of chitosan-based xerogels, dried (**a**) and immersed in pH 3.6 HCl solution (**b**) or in 10% aqueous acetic acid (**c**).

Visual aspects of the xerogels highlighted a spongy structure with a shiny pale-yellow color for CHH/T80 system and whiter, without gloss, for the citrate cross-linked sample (CHH/T80-Cit)-Figure 3a. Clove oil-loaded chitosan-based xerogel (CHH/T80/CEO) showed a yellow beige color with a slightly lighter hue after the cross-linking procedure with citrate (CHH/T80/CEO-Cit). As an overall observation, the citrate cross-linking determined the shrinking of the sample (Figure 3a) indicating a more condensed structure.

The stability of xerogels was evaluated by immersion for a long period in a mild acidic HCl solution of pH 3.6 (mimicking the gastric fluid) and in strong acidic media of 10% acetic acid (with high affinity for dissolving chitosan).

The CHH/T80 xerogel dissolved almost completely in a pH 3.6 aqueous solution but citrate crosslinking (CHH/T80-Cit) conferred the integrity of hydrogel in this media, despite some signs at the edges of the sample indicating that the limits of dispersion were approached (Figure 3b). The presence of clove essential oil in the chitosan hydrogel (CHH/T80/CEO) strongly increased the resistance to solubilization, without the need for citrate crosslinking, with only swelling observed.

The CEO-free chitosan xerogel crosslinked with citrate (CHH/T80-Cit) completely dissolved in 10% acetic acid within a few hours, without the need for stirring (Figure 3c). This was expected, considering the pH-dependent reversibility of ionic crosslinking. Surprisingly, oil-loaded xerogel only swelled, without signs of solubilization even after one month, despite the aggressive medium. This is a sign of powerful interactions between chitosan and the components of clove essential oil, conferring strong chemical resistance to the material.

**Figure 4:**
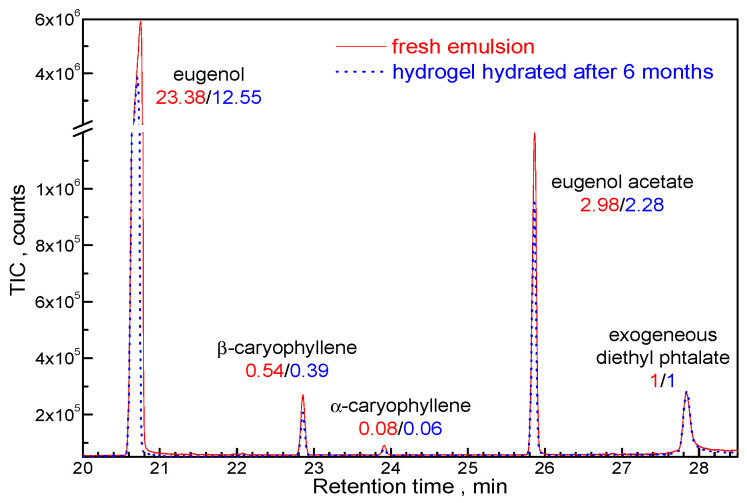
Gas chromatograms of the main organic compounds found in CHH/CEO fresh emulsion and eluted in water from the CHH/T80/CEO xerogel hydrated after six months from preparation.

The CHH/T80/CEO xerogel shelf stored for six months was immersed in distilled water at room temperature for 60 min and the released organic compounds were concentrated onto a Supelco 57328-U divinylbenzene/carboxen/polydimethylsiloxane solid phase microextraction (SPME) fiber then analyzed by gas chromatography (GC) coupled with mass spectrometry (MSD) detection. Results were compared with the SPME-GC-MSD analysis of the fresh emulsion from which the CHH/T80/CEO hydrogel was obtained. The peak areas of the identified compounds were normalized against exogenous diethyl phthalate, appearing in the GC-MSD analysis due to septum bleeding. Figure 4 shows that β/α caryophyllene and eugenol acetate were released in similar ratios compared with the initial emulsion, indicating that the availability of these bioactive compounds from CEO was not affected by xerogel storage. The strong decrease in the eugenol amount released in aqueous medium indicates that this compound could be strongly bound to the xerogel by interactions with functional groups in the chitosan macromolecular network.

### 3.2. Characterization of Xerogels and Hydrogels

#### 3.2.1. Morphology

**Figure 5:**
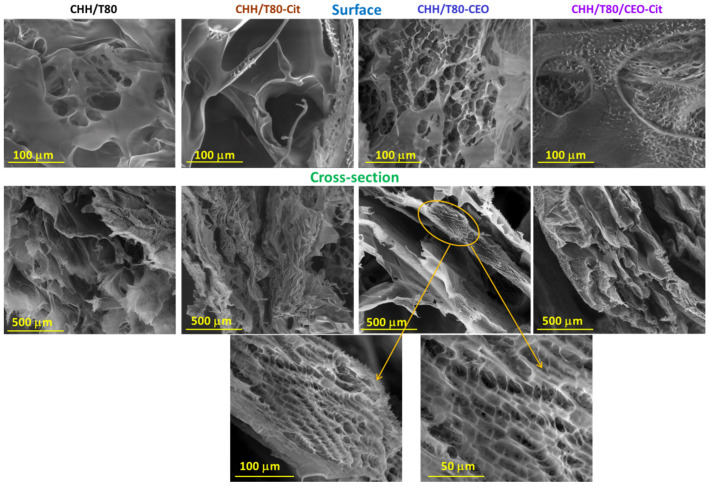
SEM images at the surface and in the cross-section of chitosan-based xerogels at different scales (in the 100–500 μm range): chitosan–Tween 80 (CHH/T80), CHH//T80 crosslinked with citrate (CHH/T80-Cit), CHH//T80 loaded with clove essential oil (CHH/T80-CEO), and CHH/T80-CEO crosslinked with citrate (CHH/T80-CEO-Cit).

In Figure 5, the SEM micrographs of freeze-dried chitosan-based hydrogels are presented. A characteristic range of pore size distribution for each sample can be noticed, which shows the porous structure of the lyophilized hydrogels. The pore size ranges varied significantly with the composition and chemical treatment. The CHH/T80 sponge showed a porous structure with smooth and thin walls, exfoliated appearance, and slightly interconnected pores. This material is brittle and with a high tendency to lose its physical integrity. Citrate solid cross-linking acts as stiffening agent for the chitosan-T80 hydrogel (CHH/T80-Cit), with the structure becoming more condensed. The SEM images revealed a highly interconnected porous structure, with increased porosity and smaller pore size. By the addition of clove oil in the chitosan-based system (CHH/T80/CEO) a super-porous material was obtained, with rough walls and fine interconnected pores. In addition, an overall regular and layered network, with macromolecules that aggregate in parallel, with a very well-defined honeycomb patterned can be noticed. This may suggest a double network, one formed by chitosan macromolecules arrangement and the second one, with a honeycomb appearance, developed by interactions with constituents in clove oil. Supplementary ionic cross-linking of the xerogel (CHH/T80/CEO-Cit) led to a decrease in pore size and an increase in the number of pores per unit area.

#### 3.2.2. FTIR and NMR Spectral Analysis

**Figure 6:**
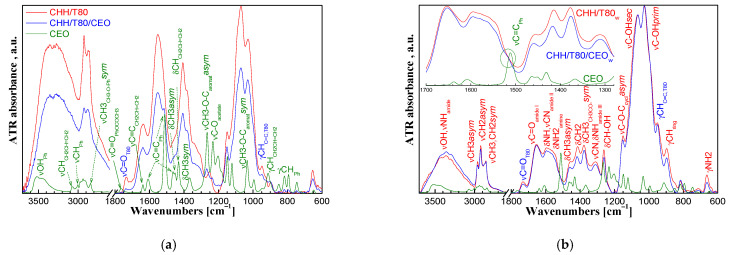
FTIR spectra of clove essential oil (CEO) and chitosan-based xerogels CEO-unloaded (CHH/T80) and CEO-loaded (CHH/T80/CEO) “as prepared” (**a**) and washed (**b**).

The FTIR spectrum of CEO was dominated by the vibration bands of the functional groups in eugenol and eugenol acetate, which are the main components of the oil (Figure 6a). The aromatic ring can be observed from the vibration bands at 3005 cm^−1^ (νCH_Ph_), 1610/1510/1465 cm^−1^ (νC=C_Ph_), and at 850/815/795/750 cm^−1^ (γCH_Ph_) while the phenolic -OH group is indicated by the wide band at 3600-3300 cm^−1^. The unsaturated bond in the allyl groups is evidenced by the νCH at 3080 cm^−1^, νC=C at 1640 cm^−1^, δCH at 1430 cm^−1^, and γCH at 910 cm^−1^. The vibration bands at 2840 cm^−1^ (νCH_3_) and at 1760 cm^−1^ (νC=O) show the methyl in methoxy groups and the ketone in acetate groups while the bands at 1265/1030 cm^−1^ (νC-O-C asymmetric/symmetric) stand for ether bonds in methoxy and acetate groups. The νC=O at 1765 cm^−1^ and the νC-O-C acetate band at 1230 cm^−1^ shows the ester group in eugenol acetate. The bands at 1450/1370 cm^−1^ correspond to the δCH_3_ asymmetric/symmetric vibrations [41].

The presence of eugenol and eugenol acetate from clove oil in CHH/CEO xerogel is shown by a shoulder at 1515 cm^−1^, the four specific peaks in the 745–850 cm^−1^ range, and the small peak at 1235 cm^−1^ corresponding to the aromatic structure and acetate functional groups. The vibration bands at 1730 and at 950 cm^−1^ corresponding to νC=O and γC=C vibrations, respectively, indicate the Tween 80 surfactant [42].

While the characterization of the “as prepared” xerogels was relevant for the intended applications of the material, valuable information on the structure was obtained after thorough washing with ethanol (by five successive immersions into 50 mL ethanol for 2 h followed by excessive rinsing with water) that removed the unbound low molecular components, leaving behind only those strongly bonded to the chains of the macromolecular network.

A large band appeared between 3700 and 3000 cm^−1^ in the FT-IR spectrum of washed chitosan-based xerogel (Figure 6b). The νOH vibrations overlapped in this range with the intense stretching vibration of the NH bond in the secondary amide groups from the acetylated units present in the chitosan structure and with the weak stretching vibration of the NH_2_ primary amine groups from the deacetylated units. The red shift of νOH vibration towards lower wavelengths of 3440 and 3360 cm^−1^ indicates the involvement of side –OH groups from the chitosan structure in intermolecular H bonds [43]. The secondary and the primary hydroxyl groups are indicated by the νC-OH vibration at 1066 and 1028 cm^−1^, respectively and by the in-plane bending (scissoring) vibration at 1260 cm^−1^ of the CH groups bond to OH.

The νC=O amide I vibration band of the acetylated groups in chitosan appeared at 1655 cm^−1^ and the δNH/νC-N amide II peak at 1595 cm^−1^ was large, covering the δNH_2_ vibration band of the amine groups from the deacetylated units. The weaker amide III vibration could be observed at 1310 cm^−1^. The primary amine groups are evidenced by the γNH_2_ vibration band at 665 cm^−1^. The band at 1150 cm^−1^ stands for the asymmetric stretching vibration of the C-O-C bridges between the saccharide rings. The CH_3_ and CH_2_ groups are indicated by the asymmetric and symmetric stretching vibrations at 2960 and 2880 and at 2923 and 2860 cm^−1^, respectively and by the asymmetric and symmetric in-plane δCH_3_ scissoring vibrations at 1460 and 1375 cm^−1^ and by the δCH_2_ vibration at 1410 cm^−1^. The γCH vibration of the saccharide ring appeared at 895 cm^−1^.

Differences could be observed in the FTIR spectra between the unloaded and CEO loaded chitosan xerogels. The central and right side of the large band at 3700–3000 cm^−1^ for overlapped hydroxyl, amide, and primary amino vibrations showed a clear increase after the incorporation of CEO into chitosan matrix. This could indicate the conversion of primary amines from the chitosan structure into secondary ones. The overlapped amide II/amino band in the 1595–1550 cm^−1^ range was smaller in the CHH/T80/CEO xerogel compared with the CHH/T80 one, while the amide I band remained unaffected. Moreover, the intensity of the γNH_2_ band was decreased by CEO incorporation into CHH/T80 xerogel. This could be explained by a smaller contribution of the primary amino groups, most probably due to their conversion into secondary amines by a reaction with compounds from the clove essential oil.

The FTIR spectrum of washed CHH/T80/CEO_w_ xerogel showed a shoulder at 1515 cm^−1^, corresponding to the aromatic rings (see details in Figure 6b). This could not be observed in the spectrum of the CHH/T80_w_ sample, indicating that eugenol and/or eugenol acetate could be chemically bonded by grafting to the chitosan macromolecular chain, most probably to the primary amino groups that were, thus, converted into secondary ones. Hence, a chitosan conjugate with the major constituents of clove essential oil is obtained. The vibration bands at 1730 and 950 cm^−1^ show that traces of Tween 80 emulsifier also remained in the material.

The effect of ultrasonic waves on the reactivity of clove essential oil in 2% *v*/*v* acetic acid solutions was evaluated by ^1^H-NMR spectroscopy using eugenol as model compound, alone or mixed with Tween 80 surfactant, recording the spectra in CDCl_3_. Chemical shifts assignments were made according to references [44,45].

Eugenol was not miscible with the acetic acid solution, but it was dispersed as fine droplets by the mechanical energy of the ultrasound waves, then it separated again at the end of sonication. An oil-in-water stable emulsion of eugenol in acetic acid was formed by adding sufficient amounts of Tween 80 emulsifier. The emulsion was destabilized after sonication, an amber colored phase that separated at the bottom of an aqueous fraction with a milky appearance indicating the partition of Tween 80 into the oily phase. The oily phases were collected and thoroughly washed several times with water and separated by centrifugation. The solution of Tween 80 in acetic acid showed no change in visual aspect after sonication.

**Figure 7:**
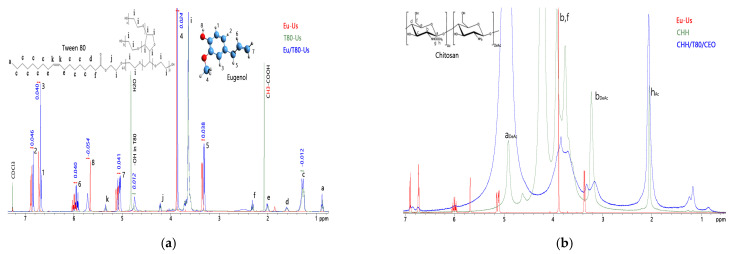
^1^H-NMR spectra of eugenol (Eu) and of Tween 80 (T80) ultrasonicated (Us) in acetic acid individually or together (**a**) and of the obtained CHH and CHH/T80/CEO hydrogels (**b**).

Changes in the chemical shift of protons in eugenol and in the Tween 80 emulsifier when mixed and sonicated were observed compared with the sonication of each compound alone in 2% acetic acid (Figure 7a). The aromatic protons and the aliphatic allyl ones in eugenol were upfielded (peaks shifted with ~0.04 ppm to lower values). It appeared that sonication stimulates the π electrons from the aromatic cycle and from the alkene bond in eugenol, shielding the nearby protons. The protons in position 4 were upfielded only with ~0.02 ppm, indicating that the low reactive methoxy group was less affected. The phenolic proton was deshielded by 0.054 ppm due to the involvement in the hydrogen bonds. On the contrary, the aliphatic protons of Tween 80 were slightly downfielded with ~0.012 ppm, while the hydroxyl ones were upfielded. The opposite shielding/deshielding of the protons indicates strong interactions between eugenol and the surfactant under the action of sonication. This explains the observed destabilization of initial Eu-T80 emulsion after sonication that allowed separate collection of eugenol fraction for NMR characterization. No separation after ultrasonication was observed for the CHH/T80/CEO emulsion used for the preparation of the hydrogels, indicating that the CEO–T80 complex interacted with the solubilized chitosan. This explains the traces of emulsifier observed in FTIR spectrum even after thorough washing of the hydrogel (see Figure 6b).

^1^H-NMR spectra were also recorded for the powdered unloaded chitosan sample solubilized in D_2_O/DCl (CHH sample) and for the chitosan–clove oil hydrogel finely dispersed in D_2_O/CD_3_COOD (CHH/T80/CEO sample). Besides the peaks of chitosan, the ^1^H-NMR spectra of the CHH/T80/CEO hydrogel (Figure 7b) had additional small peaks at 6.7–6.9 ppm, 6 ppm, 3.3 ppm, and a shoulder at 3.9 ppm, which correspond to the aromatic, unsaturated, saturated allyl and methoxy protons in the structure of eugenol, respectively. This is additional proof for the strong fixation of compounds from clove essential oil in the structure of the hydrogel.

#### 3.2.3. Rheological Properties

**Figure 8:**
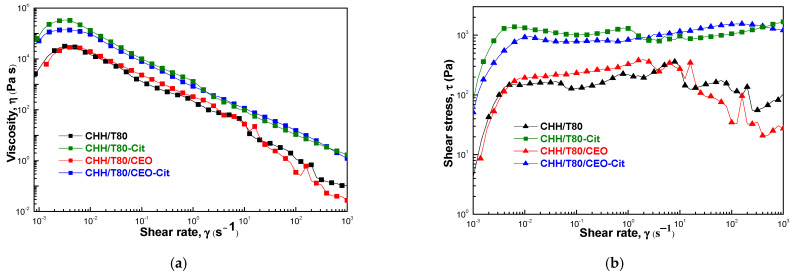
The variation of dynamic viscosity (**a**) and of shear stress (**b**) with shear rate for chitosan-based xerogels(CHH/T80), citrate cross-linked (CHH/T80-Cit) and loaded with clove essential oil (CHH/T80/CEO).

The dynamic viscosity was represented as a function of the shear rate for all studied hydrogels (Figure 8a). These manifested a non-Newtonian, pseudoplastic behavior. A slight shear thickening effect was observed for all samples at very low shear rates below ~2.5–6.0 10^−3^ s^−1^. This was followed by constant fall in the viscosity with the increasing shear rate, indicating gel-like structures. A sudden fall of viscosity with an increase in shear rate above ~10 s^−1^ for unloaded and CEO-loaded chitosan hydrogels suggests the apparition of viscous heating, which decreases the viscosity due to the heating effect caused by dissipated energy through shear. Citrate crosslinking increased the dynamic viscosity, and this was mainly due to rigidization since the viscous heating appeared at lower shear rates of ~1 s^−1^. However, loading CEO into chitosan before ionic crosslinking with citrate creates enough elasticity to absorb the shear energy without heating effect. As a result, the variation of viscosity with shear rate is monotonous, without strong variations. The viscous heating effect can be clearly observed from the shear stress-shear rate variation in Figure 8b.

**Figure 9:**
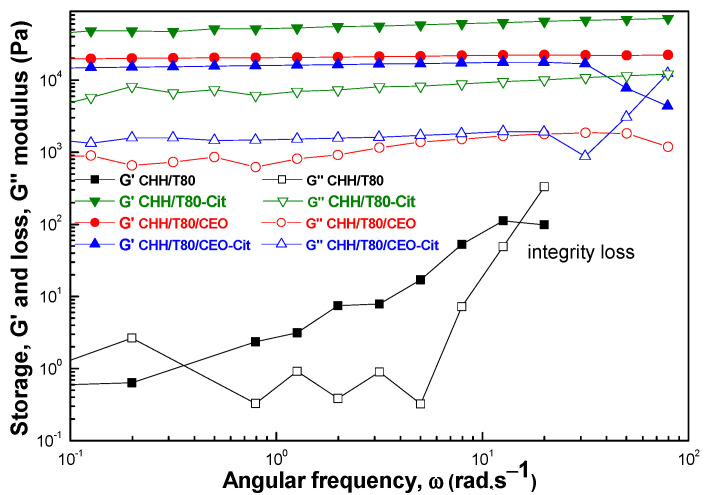
Angular frequency dependence of storage (G′, filled symbols) and loss (G″, open symbols) moduli for the chitosan-based hydrogels (CHH/T80), citrate cross-linked (CHH/T80-Cit) and loaded with clove essential oil (CHH/T80/CEO).

The viscoelastic character was evaluated from the variation of the dynamic moduli over the studied frequency range (Figure 9). Storage (G′) and loss modulus (G″), describing the elastic and viscous character of the material, respectively, were plotted as function of the angular frequency (ω) under a constant amplitude strain (LVE range-0.5%). Similar curve variations were obtained for all studied hydrogels, except for the CHH/T80 sample. The material obtained from chitosan in the presence of T80 emulsifier showed a typical weak gel, the rheological moduli, especially the loss one (G″), showing oscillation with angular frequency, as an indication that the sample was not uniform at macroscopic scale. The moduli could not be recorded at high stress, indicating a loss of physical integrity. Viscoelastic moduli were significantly increased by incorporation of CEO and by citrate crosslinking. They remained constant with the variation of angular frequency, with G′ > G″ indicating that the elastic behavior dominated the viscous one.

Crosslinking with citrate led to a rigid structure characterized by high storage and loss moduli. The clove essential oil also increased the viscoelastic moduli, the storage modulus being slightly lower (about half) than for citrate crosslinking. On the other hand, the loss modulus was one order of magnitude smaller than for citrate crosslinking, indicating a much more flexible structure, with less force being required for deformation. Sodium tri-citrate acts as a bivalent crosslinker strongly keeping together two chitosan macromolecular chains by ionic bonds with the side amino groups. It appears from the viscoelastic behavior that components of clove essential oil also behave like crosslinking agents, creating bonds that are both strong and flexible, most probably of covalent type. This is supported by the FTIR observations on the conversion of primary amines into secondary ones, as discussed above. Citrate crosslinking after the addition of CEO to chitosan weakened the strength of bonds between the macromolecular chains, G″ being slightly lower than for CEO-loaded chitosan. At the same time these bonds were less flexible due to rigidization by ionic interactions, with G′ slightly increasing.

#### 3.2.4. Swelling Evaluation

**Figure 10:**
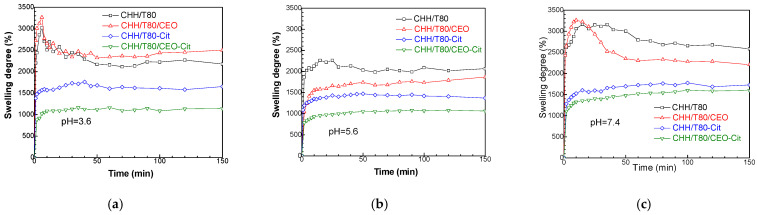
Swelling behavior of chitosan-based hydrogels (CHH/T80), citrate cross-linked (CHH/T80-Cit) and loaded with clove essential oil (CHH/T80/CEO) in various media of pH 3.6 (**a**), pH 5.6 (**b**) and pH 7.4 (**c**).

Chitosan is an amphiphilic polymer that contains both hydrophobic acetyl groups and hydrophilic amino and hydroxyl ones. The deacetylation degree of the chitosan used in this study was of 87%; therefore, the predominant amino groups induced a hydrophilic character. However, this was only moderate due to the involvement in the hydrogen intramolecular bonds with the hydroxyl groups. The pKa of native chitosan is on the slightly acidic side towards neutral (6.5, as previously determined by potentiometric titration [46]) since the protonation of amino groups is needed to equilibrate the negative ionizing hydroxyl groups which are in higher amounts in the structural units of the macromolecule. This combination of properties led to a high swelling ability (above 2000%, or 20 g of water per gram of hydrogel) of chitosan at pH 5.6. A small overshooting effect was observed (Figure 10b), the swelling degree reached a maximum value at about 25 min, followed by a slow decrease for another 25 min. down to equilibrium, which was maintained until the end of measurements (300 min, not shown). The overshooting was faster and more intense at pH 3.6 (Figure 10a), which is below the pKa of chitosan. This could be explained by the high tendency of amino groups towards protonation in strong acidic medium, leading to the rapid diffusion of water in the outer regions of the hydrogel. The rate difference between the uptake of the water from the surrounding media and its diffusion to the inner part of material creates a tension in the system (e.g., osmotic forces). This requires a relaxation of the macromolecular structure, with consequent “pumpinG″ of the water both to the inside of the material and to the outside medium, the latter resulting in a “deswellinG″ effect. Above pKa value, at pH 7.4 in Figure 10c, chitosan is deprotonated, and the swelling mainly involves the ionization of hydroxyl groups. It is well known that chitosan swelling at neutral pH is smaller than in acidic medium. However, we observed in our study that the swelling was much greater at pH 7.4 than at pH 3.6 and 5.6. It is to be considered that the reference CHH/T80 hydrogel contains an emulsifier, which acts as plasticizer, increasing the flexibility of the macromolecular structure and, therefore, the ability to uptake water. Moreover, although polysorbate 80 is considered a non-ionic surfactant, it may carry a net negative charge due to the adsorption of OH^-^ ions from water, which may determine the occurrence of electrostatic interactions with the positive charged chitosan. These interactions induce a compactness of the hydrogel network by the electrostatic attraction forces. It is also to be mentioned that the CHH/T80 sample was subjected to ultrasonication in acetic acid medium, which can produce backbone scissions, decreasing the molecular weight of chitosan. The additional terminal hydroxyl groups formed in this way improve the hydrophilicity of the material. Overshooting also occurs, this time in two steps, due to the two types of hydroxyl groups in chitosan structure, which have different availability to water molecules.

The main constituents of CEO are eugenol (85.7 wt%) and eugenol acetate (7.9 wt%) followed by α- and β- caryophyllene (summing 4.5 wt%) [37]. These are hydrophobic compounds, although eugenol and eugenol acetate have ionizing functionalities. Therefore, at pH 5.6 where equilibrium occurs between the positive and negative charges on chitosan macromolecular chains, the hydrophobic nature of CEO acts as water repellent, lowering the swelling degree, with no overshooting effect compared with reference CHH hydrogel. Similar results were obtained for a eugenol-grafted chitosan hydrogel previously reported by Jung et al. [47]. However, the ionization of eugenol and eugenol acetate occurs below and above pKa value of chitosan, affecting the swelling behavior. Protonation of methoxy and acetate groups in these compounds occurs in strong acidic media. Therefore, the overshooting effect was faster and more intense at pH 3.6, the involved additional repulsive forces increasing the distance between the macromolecules, and, as a consequence, the equilibrium swelling degree.

On the other side, the swelling behavior above pKa, at an almost neutral pH of 7.4, is intriguing, with a faster, higher, and narrower overshooting effect compared with chitosan hydrogel but with a much lower equilibrium swelling degree. This behavior cannot be explained only by the hydrophobic nature of CEO compounds and suggests a structure which is more compact but have increased flexibility.

The citrate-treated hydrogels did not contain free Tween 80 emulsifier or unbound CEO compounds, since these were removed by washing. Therefore, the swelling behavior gives intrinsic information on the macromolecular structure of these materials, without influences from free, low-molecular compounds. Citrate strongly decreased the swelling degree at all three pH values, with no overshooting effect. Moreover, no great difference was observed for the equilibrium swelling degree in strongly acidic (pH 3.6) and almost neutral (pH 7.4) medium. This was due to the rigidization of the chitosan structure by ionic crosslinking at the amino groups, decreasing protonation and chain repulsions in acidic pH media. The citrate crosslinking of CEO containing chitosan hydrogel showed the lowest swelling degree, which was slightly higher at neutral pH compared with acidic ones. This is in good agreement with the more compact structure obtained in this case, as showed by SEM images and related discussions. This suggests that a part of CEO compounds (besides the free ones in the composition of CHH and CHH/CEO samples that explained the above discussed swelling behavior) is chemically bound to the chitosan macromolecular chains. Such bonding can provide a structure which combine compactness and flexibility and can explain the swelling obs erved at pH of 7.4.

Interactions through hydrogen bonds can occur between the hydroxyl groups of chitosan and the carbonyl groups in eugenol and eugenol acetate in CEO at neutral pH, disturbing the repulsive forces between the macromolecules, with a decrease in intermolecular space and a lower ability for water uptake at equilibrium in neutral pH medium. These hydrogen bonds are disturbed in acidic pH where the protonation of nucleophile functionalities from CEO prevails, explaining the increased swelling below pKa, as previously discussed. The hydrogen acceptor functionalities in eugenol (methoxy and phenol) and in eugenol acetate (methoxy and carbonyl) can also interact with the amino groups on chitosan backbone. Therefore, there will be fewer amino groups available for protonation in acidic media, for H- bonding with hydroxyl groups on chitosan chain, or for ionic interactions with citrate, all leading to increased flexibility of the structure. On the other hand, bonding of eugenol and eugenol acetate on chitosan macromolecules through strong interactions can provide the compactness of the material. The liberated hydroxyl groups following the involvement of amino groups in bonding with CEO compounds increase the hydrophilic character of chitosan, explaining the overshooting swelling at neutral pH of 7.4.

#### 3.2.5. Antimicrobial and Antioxidant Properties

**Figure 11:**
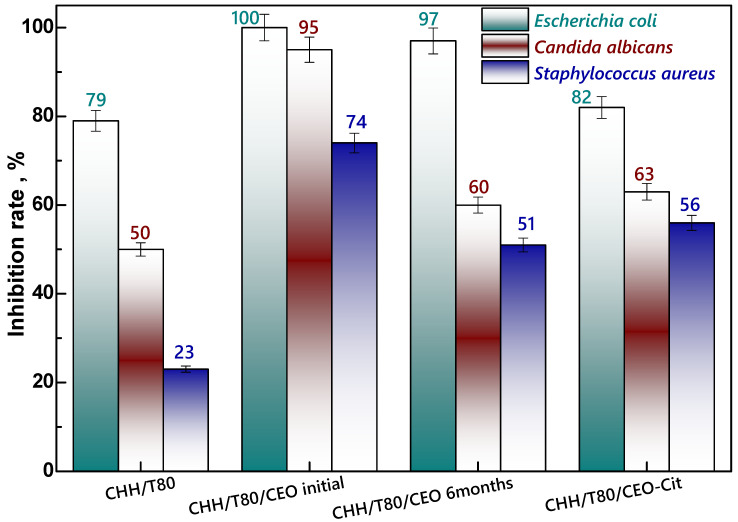
Antimicrobial activity of xerogels: unloaded chitosan (CHH/T80), clove essential oil (CEO) loaded freshly prepared (CHH/T80/CEO initial) and after 6 months storage (CHH/T80/CEO 6 months), and citrate cross-linked (CHH/T80/CEO-Cit).

The antimicrobial property of materials is a target and desirable function in many domains of applications, especially in the biomedical field (e.g., wound dressing, implants, etc.) but also in food packaging. An increase of 51% in the microbial inhibition rate against *S. aureus* was noticed by clove oil embedding into chitosan-based hydrogel (CHH/T80/CEO_initial_ in Figure 11). This result is of particular importance since this Gram-positive bacterium, *S. aureus*, is the most dangerous among all common pathogenic staphylococcal bacteria. *S. aureus* bacteraemia is a significant infection commonly for skin but which can also induce pneumonia, heart valve or bone infections, causing high rate of mortality [48].

Generally, a rather long period of time can pass from the moment of obtaining a material until its use. Thus, the antimicrobial activity, one target property of these hydrogels, was evaluated for CEO–chitosan hydrogel freshly prepared and after 6 months of shelf storage. The CEO-induced effect decreased only moderately after 6 months, more exactly from 74% inhibition of initial sample (CHH/T80/CEO_initial_) to 51% after 6 months (CHH/T80/CEO_6 months_). The maintained effect was still significant, suggesting a successful embedding of CEO into the chitosan matrix with preserved functionality. Citrate cross-linking of the CEO-chitosan hydrogel (CHH/T80/CEO-Cit) determined a moderately decrease in the antimicrobial activity against all the three bacteria tested. The most pronounced reduction, of about 32%, was observed on *Candida albicans*, which is a Gram-positive fungus, when compared with the citrate-free cross-linked sample (CHH/T80/CEO). This result indicates that citrate-crosslinking mainly reduce the antifungal activity and less the antibacterial one. However, the citrate cross-linked CEO embedded xerogel had a similar antibacterial activity to the sample after six months and yet still higher than the chitosan reference sample (CHH/T80). Ageing of CEO-loaded sample affected in a similar manner as citrate cross-linking did the antimicrobial activity against the *C. albicans* fungus. The greatest gain of combining clove oil with chitosan is represented by the fact that CEO loading into hydrogels enhances the antimicrobial activity of the chitosan, in particular against *Staphylococcus aureus* bacterium.

**Figure 12:**
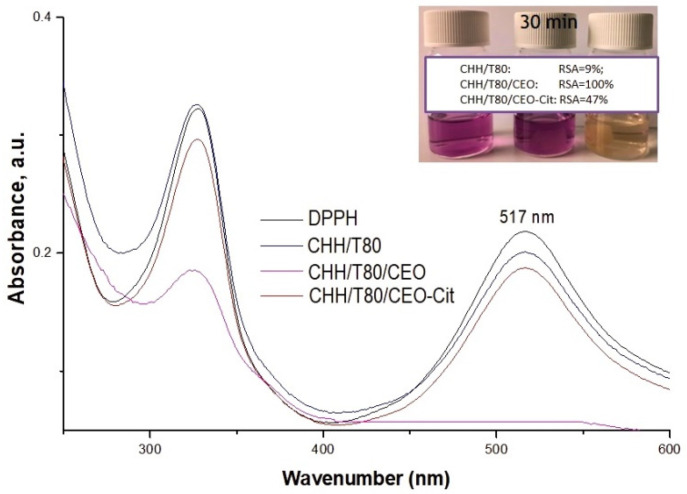
UV-Vis spectra and photographs of DPPH solutions free or incubated with chitosan-based xerogels (CHH/T80), loaded with clove essential oil (CHH/T80/CEO) and citrate cross-linked (CHH/T80/CEO-Cit).

The assessment of the antioxidant potential of the chitosan-based hydrogels was achieved by determining their inhibition capacity of free DPPH radicals, which was evaluated after 30 min of incubation in dark-Figure 12. The unloaded chitosan-based xerogel (CHH/T80) exhibited a slight scavenging potential towards DPPH radical, calculated as about less than 10%. This is in good agreement with previous observations of Xia et al. [49] that found that CEO in situ addition into the chitosan-based system has conferred to xerogel material strong antioxidant activity. A total radical scavenging activity (RSA of 100%) was noticed for this sample, color fading appearing in less than 5 min from the immersion of 16 mg xerogel into 6 mL of 0.05 mM DPPH solution. Citrate cross-linked CEO-loaded chitosan xerogel exhibited a lower antioxidant activity of about 47%. This may be due to the fact that the strong cross-linking of the network delays the release of active compounds into the liquid media but also to the supplementary washing steps involved into the protocol of solid cross-linking with citrate. The 6 month aging of the CEO-loaded sample does not affect the radical scavenging ability.

## 4. Conclusions

Clove essential oil-loaded chitosan xerogels with two-level ordered porosity and nano-structured network walls were obtained, with pronounced compactness and flexibility, pH sensitive swelling behavior, and antioxidant and improved antimicrobial activity. The morphology of the materials along with the rheological properties greatly influenced the water uptake ability of chitosan-based xerogels. The antioxidant activity of xerogels was not lost after six months of storage, while antimicrobial activity was only marginally affected. Ultrasound assisted emulsification was proved to be an efficient method for the in situ loading of hydrophobic bioactive agents into hydrophilic polysaccharide-based hydrogels. It was found that compounds from clove essential oil strongly interacted with chitosan, providing a very good chemical stability of hydrogels in aggressive acidic media, without the need for classical chemical cross-linkers.

## Data Availability

Not applicable.
